# Composition and Functional Properties of the Edible Spear and By-Products from *Asparagus officinalis* L. and Their Potential Prebiotic Effect

**DOI:** 10.3390/foods13081154

**Published:** 2024-04-10

**Authors:** Isabel Goñi, Alejandra García-Alonso, Claudio Alba, Juan Miguel Rodríguez, María Cortes Sánchez-Mata, Rafael Guillén-Bejarano, Araceli Redondo-Cuenca

**Affiliations:** 1 Department of Nutrition and Food Science, Complutense University of Madrid, 28040 Madrid, Spain; igonic@ucm.es (I.G.); c.alba@ucm.es (C.A.); jmrodrig@vet.ucm.es (J.M.R.); cortesm@ucm.es (M.C.S.-M.); arared@ucm.es (A.R.-C.); 2 Phytochemicals and Food Quality Group, Instituto de la Grasa, Spanish National Research Council (CSIC), 41013 Sevilla, Spain; rguillen@ig.csic.es

**Keywords:** asparagus, by-products, nutritional composition, microbiota, bioactive compounds, functional properties

## Abstract

Asparagus is a healthy food appreciated for its organoleptic characteristics, nutritional composition and physiological properties. During its industrial processing, a large amount of by-products are generated, since only the apical part of the vegetable is considered edible and a large amount of by-products are generated that could be of nutritional interest. Therefore, the nutritional composition of the edible part and the two by-products of the plant (root and stem) was evaluated, including dietary fiber, inulin, low-molecular-weight carbohydrates, low-molecular-weight polyphenols and macromolecular polyphenols. The hydration properties, oil retention capacity, glucose retardation index and impact on bacterial growth of both probiotic bacteria and pathogenic strains were determined. All samples were high in fiber (>22 g/100 g dw), fructans (>1.5 g/100 g dw) and polyphenolic compounds (>3 g/100 g dw) and had good water-, oil- and glucose-binding capacity. In addition, they promoted the growth of probiotic strains but not pathogenic ones. The effects were more pronounced in the spear by-product samples and appear to be related to the components of dietary fiber. The results indicate that edible spear has potential beneficial effects on host health and microbiota when ingested as part of a healthy diet, while the by-products could be used as supplements and/or as natural ingredients in fiber-enriched foods that require emulsification and are intended to achieve a prebiotic effect.

## 1. Introduction

Spain is one of the world’s leading producers of green asparagus (*Asparagus officinalis* L.). It is a food of excellent nutritional and culinary quality. The asparagus plant can be divided into three portions; the edible portion represents 24% of the plant. The remaining 76% corresponds to a hard stem and root, which are both considered to be by-products. During industrial processing, a large amount of waste is generated. The nutritional composition, bioactive compounds content, properties and applications of asparagus have been recently reviewed [1]. Both the food and its by-products have been extensively studied as sources of nutrients and bioactive compounds, many of them associated with the fiber matrix. They could be added as functional ingredients, and they could present interesting health-promoting properties.

Moreover, the current market offers a good opportunity for the development of fiber-rich foods, as dietary fiber intake is deficient in Westernized countries. However, the essential role of fiber in maintaining health is widely known [2]. Therefore, one way to increase intake could be through foods with a high-quality fiber content. Therefore, sources of fiber from fruits and vegetables are seeing increased demand, as they are of high nutritional quality. Asparagus is a good source of dietary fiber that is rich in fructans and polyphenolic compounds. All these components can interact with the colonic microbiota and modify its composition [3] and, therefore, may act as prebiotics, which are defined as substrates that are selectively utilized by host microorganisms, conferring a health benefit [4]. Prebiotics may also stimulate the colonization and activity of probiotic microorganisms, which are defined as live microorganisms that, when administered in adequate amounts, confer a health benefit on the host [5]. 

This study has focused on measuring, on the one hand, the proximal composition, minerals, non-digestible carbohydrates and polyphenolic compounds of the edible part and non-edible by-products of green asparagus, var. *Primens*. On the other hand, the functional properties related to water, oil and glucose retention have been studied, together with the possible effect on the stimulation of the proliferation of probiotic bacteria and the effect on the control of the growth of pathogenic strains.

## 2. Materials and Methods

### 2.1. Materials

Asparagus (*Asparagus officinalis* L., var. *Primens*) were collected in Chipiona, Cadiz, Spain. Each freshly collected asparagus was cut into three parts: the edible portion, corresponding to the aerial end of the asparagus (15 cm in length), and two by-products, one of them being the rest of the spear, which was around 15–18 cm length (hard spear by-product) and the other being the root (root by-product).

Samples were freeze-dried (Freeze-dryer Telstar, mod. Cryodos, Spain), blended and homogenized by grinding to a fine powder to pass through a 1 mm sieve and were stored at 4 °C prior to analysis.

### 2.2. Proximate Composition

All determinations were analyzed according to the AOAC methodology [6]. Moisture was determined by the oven-drying method at 105 ± 1 °C. The protein was determined by the Kjeldahl method using 6.25 as the conversion factor to transform the quantified nitrogen into protein (920.87 AOAC method [4]). The fat content was measured in a Soxhlet system by extraction in diethyl ether solvent (920.39 AOAC method [6]). Total carbohydrates were quantified by spectrophotometric analysis using anthrone as the reagent and glucose as the standard according to the method described by James [7]. This method was adapted to a Synergy HTX Absorbance Microplate Reader (BioTek Co., SA Winooski, VA, USA). Sucrose, glucose and fructose were analyzed by HPLC (see Section 2.5). The enzymatic-gravimetric AOAC method was carried out to determine the total dietary fiber (TDF) [8]. TDF were isolated by sequential enzymatic hydrolysis with heat-stable α-amylase (pH 6, 100 °C, 30 min), protease (pH 7,5, 60 °C, 30 min and amyloglucosidase (pH 4,5, 60 °C, 30 min). Ash content was determined by incineration of samples at 550 °C in a muffle furnace (930.22 AOAC method [6]).

### 2.3. Mineral Composition

Dried samples were incinerated in oven at 450 °C until complete mineralization, according to method 930.05 of AOAC [4], with slight modifications. Then, the ashes were dissolved with 2 mL of 50% HCl + 2 mL 50% HNO_3_, filtered and distilled water was added, resulting in a final measurement of 50 mL. Solutions were subjected to mineral elemental analysis through Atomic Absorption Spectrometry using an air/acetylene flame in the conditions of slit and wavelength recommended for each individual element. For Ca and Mg determination, a 1/10 dilution with LaCl_2_ (1.8%, w/v) was performed; for Na and K analysis, solutions were also diluted to 1/10 with CsCl_2_ (0.2%, w/v); the aim of this was to avoid interferences. Calibration lines for each element were built through the measurement of different standard solutions with the known content of each element assayed.

### 2.4. Dietary Fiber Monosaccharides and Uronic Acids

Dietary fiber was also determined according to the Englyst protocol [9]. Neutral sugars quantification was performed by gas liquid chromatography (GLC) in a Perkin-Elmer Autosystem Chromatograph equipped with a hydrogen flame ionization detector, using β-d-allose (Fluka) as the internal standard. The uronic acids were determined spectrophotometrically according to the colorimetric method of 3,5-dimethylphenol [10] adapted to microplate with a Synergy HTX Multi-Mode (Bio-Tek Instruments, Winooski, VT, USA).

### 2.5. Inulin and Low Molecular Weight Carbohydrates by HPLC

Sample extracts were obtained using a solution of 1 g of lyophilized sample in 40 mL of hot distilled water. The solution was kept in a water bath at 70 °C and in constant agitation for 1 h. Then, it was filtered before the HPLC assay was performed. Determination of inulin and low-molecular-weight carbohydrates (LMWC) was conducted by liquid-chromatography analysis using an Agilent 1100 Series HPLC, equipped with a refractive index detector (RID) on a RezexTM ROA-Organic Acid H+ (8%), 300 mm × 7.8 mm column, protected with a Carbo-H 4 × 3.0 mm ID security guard cartridge (Phenomenex España, Madrid, Spain). Ultrapure Milli-Q water (Milli-Q Integral 5 Water Purification System from Millipore) acidified with 2.5 mM H_2_SO_4_ was used as mobile phase, at a flow rate of 0.4 mL/min. The column was maintained at a constant temperature of 25 °C. Inulin and different LMWC standards (DP4 = stachyose, DP3 = 1-Kestose, DP3 = raffinose, DP2 = cellobiose, DP2 = sucrose, DP1 = fructose and glucose) were used for calibration. Regression standard curves were obtained for concentration versus area (mV × s).

### 2.6. Determination of Phenolic Compounds and Saponins

#### 2.6.1. Low Molecular Polyphenols (LMP) and Macromolecular Polyphenols (MP)

##### Extraction

Samples were previously extracted following the methodology described by Saura-Calixto & Goñi [11]. Briefly, samples were sequentially extracted at room temperature with a solution of methanol/water in acid medium and acetone/water. Each solid sample (0.5 g) was placed in a capped centrifuge tube, 20 mL of acidic methanol/water/HCl (50:50, *v*/*v*; pH 2) was added and the tube was thoroughly shaken at room temperature for 1 h. The tube was centrifuged at 2500 g in a Thermo Heraeus Megafuge 11 (Thermo Fisher, Waltham, MA, USA) for 10 min and the supernatant was recovered. Then, 20 mL of acetone/water (70:30, *v*/*v*) was added to the residue, and shaking and centrifugation were repeated. The methanol and acetone extracts were combined and used to determine LMP. Residues from the extraction were used to determine MP.

##### Analysis

LMP were determined by the Folin–Ciocalteau procedure [12]. The results were expressed as mg of gallic acid equivalents per g of dry matter.

Residues from the double extraction were subjected to two different previously reported procedures [13] in order to determine the main macromolecular polyphenols, proanthocyanidins (MPPs) and hydrolysable polyphenols (MHPs). Extraction and analytical procedures were performed in triplicate. The results were expressed as mean values ± standard deviation, on a dry matter basis.

To determine the MPPs, residues (200 mg) from the methanol/acetone water extraction were treated with 5 mL/L HCl-butanol (3 h, 100 °C) [14]. MPPs were calculated from the absorbance at 550 nm of the anthocyanidin solutions. Condensed tannins from Mediterranean carob pod (*Ceratonia siliqua* L.) supplied by Nestlé S.A. (Vevey, Switzerland) were treated under the same conditions to obtain standard curves. The results were expressed as g per 100 g of dry matter.

MHPs comprise hydrolysable tannins, phenolic acids and hydroxycinnamic acids that are released from the food matrix by strong acidic hydrolysis. To determine the MHPs, residues from the methanol/acetone water extraction were treated with methanol/H_2_SO_4_ 90:10 (*v*/*v*) at 85 °C for 20 h [15]. The hydrolysate was recollected for polyphenols analysis with Folin–Ciocalteau reagent [12]. These methods were adapted to a Synergy HTX Absorbance Microplate Reader (BioTek Co., SA Winooski, VA, USA). The results were expressed as mg of gallic acid equivalents per g of dry matter.

#### 2.6.2. Determination of Phenols and Saponins by HPLC

##### Extraction

Phenols and saponins were both detected and quantified within the ethanolic extracts derived from various samples. The ethanolic extraction process utilized a mixture of ethanol and water (80:20, *v*/*v*) in a ratio of 1:40 (dry weight to volume). This mixture was thoroughly homogenized using an Ultraturrax device (UltraTurrax T25, Janke & Kunkel/IKA Labortechnik, Munich, Germany) for a duration of 1 min at maximum speed and then filtered through a paper filter. The remaining residue underwent an identical extraction procedure. The resulting ethanolic extracts were combined and subsequently preserved at a temperature of −20 °C until they were subjected to analysis. It is worth noting that all extractions were conducted in duplicate.

##### Analysis of Phenolic Compounds by HPLC-DAD

Flavonoids and phenolic acids were quantified utilizing a Jasco-LC-Net II ADC liquid chromatograph system manufactured by Jasco (Madrid, Spain), and equipped with a Diode Array Detector (DAD), as outlined by Hamdi et al. [16]. The separation process took place within a Mediterranea Sea C18 reverse-phase analytical column (25 cm in length and 4.6 mm in internal diameter, with a particle size of 5 µm), which was provided by Teknokroma (Barcelona, Spain). To achieve separation, an elution gradient was employed, incorporating two solvents, A (water with 1% formic acid) and B (acetonitrile with 1% formic acid). The solvent B proportion was incrementally adjusted, commencing at 0% and reaching 15% over the course of 10 min, followed by a 5 min maintenance period at 15%. Subsequently, it was raised to 20% over the next 10 min, maintained at 20% for 5 min, further increased to 100% over the subsequent 5 min, held at 100% B for 5 min and ultimately returned to the initial conditions over the following 5 min. The column outlet was directly linked to a Diode Array Detector (DAD), specifically the MD-2018Plus model by Jasco. Spectra for all peaks were recorded within the 200–600 nm wavelength range, and the chromatograms were acquired and quantified at 360 and 280 nm.

The quantification of individual flavonoids was directly conducted by constructing an eight-point regression curve covering a range from 0 to 250 μg/mL, utilizing standards as the quantification basis. The final results were determined by averaging the data from two separate replicates.

##### Analysis of Saponins by HPLC-MS

The assessment of saponin content was conducted in accordance with the methodology outlined by Vazquez-Castilla et al. [17,18]. An HPLC system manufactured by Waters Alliance (Manchester, UK) was utilized, coupled with a Mediterranea Sea C18 reverse-phase analytical column (25 cm in length and 4.6 mm in internal diameter, with a particle size of 5 µm), provided by Teknokroma (Barcelona, Spain). For the separation, an elution gradient was employed, involving two solvents, A (water with 1% formic acid) and B (acetonitrile with 1% formic acid). The gradient proceeded as follows: 0–30 min with 20% B, 30–60 min with a linear gradient to 30% B, 60–70 min with a linear gradient to 100% B, and finally, 70–80 min with a linear gradient back to 20% B. The column flow was maintained at 1 mL/min. Saponin detection was achieved using an online-connected quadrupole mass analyzer (Waters Acquity QDa Detector, Waters Inc., Manchester, UK). Electrospray ionization (ESI) mass spectra were acquired at ionization energies of 50 and 100 eV in negative mode and 50 eV in positive mode, with scans ranging from m/z 200 to 1200. Specific instrument settings included a capillary voltage of 3 kV, a desolvation temperature of 200 °C, a source temperature of 100 °C and an extractor voltage of 12 V.

To establish calibration curves, two distinct external standards, protodioscin and shatavarin IV, were employed. For each standard, ten dilutions from 0 to 500 µg/mL were prepared. The selected ion chromatogram corresponding to the molecular ion of each standard in negative mode at 50 eV was integrated, and the resulting peak areas were plotted against the concentration, followed by regression analysis.

### 2.7. Functional Properties

#### 2.7.1. Swelling

The method involves the dispersion of a known weight of dry sample in a volume of water in a measuring cylinder and the measurement of the volume occupied by the hydrated fiber after 18 h. Briefly, 250 mg of sample was weighed into a graduated measuring cylinder (0.1 mL graduations) and 10 mL of distilled water was added. The sample was dispersed with gentle stirring and left on a level surface overnight at room temperature to allow the sample to settle. The volume (mL) occupied by the hydrated sample was recorded. Swelling was expressed as mL per g of dry sample. Determinations were made in triplicate.

#### 2.7.2. Water-Holding Capacity (WHC)

The method involves hydrating a known weight of sample, subjecting it to a centrifugal force to allow the excess supernatant to drain from the pellet, and recording the weight of water retained by the sample. Briefly, the sample (250 mg) was weighed in a pre-weighed 50 mL centrifuge tube and 30 mL deionized water was added. The sample was shaken to aid dispersion and hydration during 1 h at room temperature. Finally, the sample was centrifuged (3000 r.p.m, 20 min, 25 °C). The supernatant was discarded and the residue was weighed and dried at 100 °C until reaching a constant weight. Water retention was expressed as g water retained per g dry sample. The number of determinations was >3.

#### 2.7.3. Oil-Holding Capacity (OHC)

The methodology followed in this determination was similar to that previously described for WHC, using olive oil instead of distilled water. Results were expressed as g oil retained per g dry sample. The number of determinations was >3.

#### 2.7.4. Glucose Retardation Index (GRI)

The procedure of Goñi et al. [19] was followed. Dialysis bags 17 cm in length (size 9–91.4/81.3 cm Medicell International Ltd., London, UK) were thoroughly hydrated and filled with 15 mL of glucose solution (2 g/L) mixed thoroughly with 100 mg sample. Two control bags were included in each trial, one with glucose, but without sample, and the other with sample, but without glucose. Citrus pectin and guar gum were also used as reference samples. Each bag was suspended in 80 mL of distilled water in a separate magnetically stirred bath at 37 °C for 60 min. Aliquots of the dialysate were collected and the glucose concentration was determined using a glucose oxidase assay. The complete test (study with samples, positive and negative controls and reference materials) was repeated 4 times.

GRI was calculated as a percentage of glucose retained by the sample to respect a negative control with glucose alone. The glucose retardation index was calculated as indicated below:

GRI = 100 − (glucose diffused in presence of sample/glucose diffused in membrane without sample × 100)

### 2.8. Evaluation of the Prebiotic and Antimicrobial Activities of the Asparagus Fractions

A total of 20 bacterial strains belonging to 14 bacterial species were used to assess the effect of the asparagus fractions (spear edible portion, spear by-product and root by-product) on their growth. All of these species may inhabit or travel through the human digestive tract. Some of the strains (*n* = 10) belong to species that are considered as beneficial for the host: *Bifidobacterium animalis* MP320, *Bifidobacterium breve* MP297, *B. breve* MP307, *Bifidobacterium longum* MP324, *Lacticaseibacillus rhamnosus* GG, *Lactiplantibacillus plantarum* MP303, *Ligilactobacillus salivarius* MP98, *L. salivarius* MP100 and *Limosilactobacillus reuteri* MP301. The rest of the strains (*n* = 10) were isolated from cases of food poisoning, including Gram-positive bacteria (*Bacillus cereus* MP383, *Listeria monocytogenes* MP377, *L. monocytogenes* MP380, *Staphylococcus aureus* MP364, *S. aureus* MP369) and Gram-negative bacteria (*Escherichia coli* MP386, *E. coli* MP388, *Klebsiella pneumoniae* MP390, *Salmonella enterica serovar enteritidis* MP395 and *S. enterica serovar typhimurium* MP399). All the strains belonged to the collection of the research group UCM920080 (Complutense University of Madrid, Spain).

The lactic acid bacteria and bifidobacteria strains were grown in MRS broth (Oxoid, Basingstoke, UK) supplemented with L-cysteine (2.5 g/L) (MRS-C), which were incubated anaerobically (85% nitrogen, 10% hydrogen, 5% carbon dioxide) within an anaerobic workstation (DW Scientific, Shipley, UK), at 37 °C for 72 h. The remaining strains were inoculated in BHI broth (Oxoid), which was incubated in aerobiosis at 37 °C for 48 h. The initial concentration in the broth tubes was similar (~5 log10 colony-forming units (CFU)/mL) for all the strains tested in this study. Four tubes were prepared for each strain. The first was inoculated with 0.1 g/L of the freeze-dried edible fraction of the asparagus, while the second and the third ones were supplemented with the same quantity of the non-edible and root fraction, respectively. The fourth tube was not supplemented and served as a control. Tubes containing one of the asparagus fractions but without inoculating the strains were also included to assure that there was no bacterial growth due to contaminations of the asparagus-derived material. After the incubations, serial decimal dilutions of the cultures were performed using sterile peptone water and bacterial enumeration was carried on MRS-C plates (for lactic acid bacteria and bifidobacteria) or on BHI plates (for the other strains). The incubation conditions of the plates were identical to those described for the respective broth media. These assays were repeated five times per strain and per asparagus fraction.

### 2.9. Statistical Analysis

Analyses were performed in triplicate and data were presented as (g/100 g dry basis) mean ± standard deviation (SD). To establish the statistical significance of differences (*p* < 0.05), unifactorial ANOVA was applied, and Duncan’s Multiple Range Test. SAS version 9. software was used for this purpose. The correlation analysis among DF fractions and functional properties was performed with Pearson’s correlation test.

Microbiological data were recorded as CFU/mL and transformed to logarithmic values before statistical analysis. An analysis of variance (ANOVA) was conducted to examine the overall differences in bacterial concentrations across the different asparagus fractions. The assumptions of the ANOVA were verified, and data were found to meet the requirements of normality and homogeneity of variance.

Post hoc pairwise comparisons were made using Tukey’s test to examine specific differences between the different asparagus fractions [20]. The resulting *p*-values were adjusted for multiple comparisons to control the family-wise error rate.

To provide an intuitive representation of the significant differences among the asparagus fractions, mean concentration values were allocated into different letter groups. Those fractions sharing the same letter did not significantly differ according to Tukey’s test (*p* < 0.05), following the methods described by Piepho [21].

Before carrying out any additional analysis, the data underwent a preprocessing stage, which was executed using the R programming language [22]. To standardize each variable, autoscaling was implemented, ensuring each had a mean of 0 and a standard deviation of 1. Also, any values that were detected as falling below the limit were replaced with half the value of the detection limit.

After this preprocessing, a cluster analysis was performed. The Gap Statistic method, a commonly accepted method for discerning the ideal number of clusters in a dataset [23], was used. The ‘factoextra’ package in R [24] was utilized to determine the optimal quantity of clusters.

After completing the cluster analysis, a principal component analysis (PCA) was carried out. This analytical step was also performed using the ‘factoextra’ package in R [24]. The PCA allowed us to gain a detailed understanding of the dataset’s inherent structure, facilitating the detection of patterns and associations. In line with this analysis, confidence ellipses were generated in the PCA bi-plot, with each one encompassing 75% of the samples within its corresponding cluster.

## 3. Results and Discussion

### 3.1. Proximate Composition

Table 1 shows the results of the proximal composition of the three parts of the asparagus analyzed expressed as g/100 g on a dry matter basis. The moisture was lowest in the root zone (59.00 g/100 g), while the basal stem by-product and spear edible portion showed similar values close to 90.00 g/100 g. Redondo-Cuenca et al. [25] found values of 61.25 g/100 g dry matter for roots, 86.25 g/100 g for spear by-product and 88.03 g/100 g for edible portion in a green asparagus *Primens* variety. Root and spear edible part moisture values were also in agreement with those found by Adouni et al. [1] and Palfi et al. [26], respectively.

In the macronutrient content, all samples stand out for their low fat content of less than 4 g/100 g, which has the highest value in the edible part of the spear (3.39 g/100 g, compared to 1 g/100 g in the root). Similar values for the edible part were reported by Aberoumand [27] and Palfi et al. [26]. This makes all parts of asparagus of interest for low-fat diets. Regarding their protein content, asparagus showed a remarkable contribution of this macronutrient, being significantly higher in the edible part with 48.82 g/100 g compared to the hard stem by-product with 14.96 g/100 g, while the root presented an intermediate value of 17.80 g/100 g. This trend agrees with Redondo-Cuenca et al. [25] and Adouni et al. [1].

In the case of carbohydrates, asparagus is characterized by variable carbohydrate values depending on the time of ripening, and these are also dependent on the crop and the type of variety [28]. In the case of the *Primens* variety, it has been observed that the values of the digestible fractions, total carbohydrates and the non-digestible, mainly dietary fiber, were higher in the root and stem by-products, as shown in Table 1. Specifically in the total carbohydrates of the three asparagus fractions analyzed, it was observed that the edible zone had the lowest contribution of this macronutrient compared to the by-product, with values of 17.27 and 28.68 g/100 g, respectively, and as for the root, the content was much higher (32.17 g/100 g). The values of Redondo-Cuenca et al. [25] showed a similar trend, but they reported somewhat higher values of these carbohydrates related to the difference in the variety studied.

Concerning the content of free sugars, disaccharides and monosaccharides, the sucrose content was significantly higher in the root than in the other two areas of the asparagus. As for monosaccharides, reducing sugars (glucose and fructose) were higher in the spear by-product and in the edible part with a proportion of 52.3% and 85.3% of the total, respectively, indicating that sugars such as glucose and fructose were found in a higher proportion of free sugars compared to the root values, where reducing sugars represented 8.3% of the total carbohydrates. In the root, these sugars are possibly found to form oligosaccharides or polysaccharides, probably as a reserve substance. Soteriou et al. [29] showed similar results for sucrose, glucose and fructose that we found in the edible part of green asparagus in our work. Bhowmik et al. [30] indicated that the main carbohydrates found in the edible part of asparagus are fructose and glucose, with a minor amount of sucrose. It has been seen in previous studies that the total sugar content in asparagus also showed a spatial distribution, with the highest content in the lower part and the lowest in the tip portion [28,31], which agrees with the values of the present study.

With respect to non-digestible carbohydrates, dietary fiber presented high values in all the samples, although they reached a much higher amount in the two by-products, as was to be expected due to the part of the plant to which they correspond. Thus, the TDF in the by-products was 57.70 and 40.52 g/100 g in the spear by-product and root, respectively, while in the edible part, dietary fiber was lower (22.85 g/100 g).

As for the content of minerals, the analysis of ashes showed similar values in the by-products, close to 5 g/100 g, while in the edible part the values were 9.34 g/100 g. According to other authors and as in the case of sugars, there is a spatial distribution of minerals in the asparagus spear in such a way that the highest concentration of minerals occurs in the apical portion, probably due to the greater cell growth and development in this portion [32]. Table 2 shows the different minerals that make up these ashes.

In general, the results of the present study regarding the composition of the edible part of asparagus were like those shown by Chitrakar et al. [28] and Redondo-Cuenca et al. [25] with slight differences regarding protein and total carbohydrates. The former authors state that asparagus spears and stem by-products are rich sources of nutritional compounds and phytochemicals, the content of which is affected by different factors, including asparagus variety, spear part, harvesting season, cultivation method, etc.

### 3.2. Minerals

As it can be seen in Table 2, the analyzed samples of asparagus by-product had total mineral contents around 5 g/100 g dry matter, while the edible portion of the spear presented much higher content (9.3 g/100 g dry matter). It has been previously reported that there is a spatial distribution of minerals in asparagus spear, with the highest concentration of minerals occurring at the apical portion, probably due to higher cellular growth and development in this portion [28,32].

The macroelements K, Na, Ca and Mg were quantified in all the samples, as well as Fe as a major microelement. Zn was only detected in low amounts in root by-product and Cu and Mn were not detected in any of the products analyzed. The amounts of minerals in all samples followed the sequence K > Ca > Mg > Na > Fe, in agreement with a previous study [25] on *A. officinalis*, var. *Herkolim* edible portion and by-products.

Adouni et al. [1] studied mineral contents in the root and rhizomes of *A. stipularis*; the values found in the present work for root by-product of *A. officinalis, var Primens* were slightly lower for Mg, similar for Ca and much higher for K than values reported by these authors. However, for samples of A. officinalis analyzed by Motoki et al. [33], the Ca and Mg content in the roots was similar to those found in this study.

On the other hand, when comparing with data from the edible portion reported in other studies, it can be seen that the values of Na and Mg found in this study were similar to those reported by Motoki et al. [33] and Moreiras et al. [34], while other elements were found in lower amounts. The analyzed edible spears also presented values of Mg similar to those reported by Amaro-López et al. [32] for spears of *A. officinalis, cv. Mary-W* and *UC-150.*

It has to be remarked that K showed an increase from the root to the spear of the plant, in agreement with the findings reported by Amaro et al. [32] and Redondo-Cuenca et al. [25]. These authors also reported that other minerals did not show an increasing or decreasing trend in the different parts of the plant, as is shown in this study. The statistical study applied to the results found in the present study evidence that Ca and Fe was significantly higher (*p* < 0.05) in root by-product, while K and Mg presented significantly higher amounts (*p* < 0.05) in the edible portion of the spear; on the other hand, spear by-product stood out with the lowest Na content, although this mineral presented low content in all the samples analyzed.

According to the results found in this study, it could be stated that asparagus spear edible portion and root-by product, as a dry ingredient, may be a good source of K, Ca and Mg to be used for food enrichment. When comparing to EFSA [35] Dietary Reference Values (DRV) for the adult population, it can be found that a portion of 100 g of these ingredients could provide 24–37% of calcium DRV, more than 42–77% of potassium DRV and 31–60% of magnesium DRV, which could offer an interesting contribution for the daily intake of these minerals in the diet.

### 3.3. Dietary Fiber Composition

Table 3 shows the results related to the monomeric composition of DF of the analyzed green asparagus fractions expressed as dry matter. Of the three green asparagus fractions studied, the spear by-product stands out for its higher TDF NSP content (43.10 g/100 g), followed by the root by-product (29.66 g/100 g) and finally the edible portion of the spear (13.70 g/100 g). The edible portion of the shoot is noteworthy due to the higher proportion of uronic acids (28%) compared to the total neutral sugars (72%). In the case of the root by-product, the presence of uronic acids is the lowest of the three parts studied (10%). In the three materials, xylose and glucose stand out, especially in the spear by-product, which indicates a greater amount of cellulose and xylan-type hemicelluloses. Jaramillo-Carmona et al. [36] studied the green asparagus spear by-product and indicated that glucose was the majority sugar highlighting the presence of a high percentage of cellulose in this material, and xylose and arabinose, as arabinoxylans were also postulated as the main hemicelluloses in the cell walls of green asparagus along with xyloglucans. In the present work, the amount of arabinose was much lower than that of xylose and glucose, as can be seen in Table 3. The profile in neutral sugars and uronic acids of TDF NSP of the materials studied clearly corresponds to the type of portion, thus in the edible part, the youngest and growing tissue, a smaller difference can be observed between each of the neutral monomers and a greater presence, in proportion, of uronic acids that mostly comprised pectic substances, characteristic of this type of tissue. In the same way, the profile of spear by-product corresponds to an aged part where cellulose and lignin predominate. When comparing these results with those obtained in green asparagus of the *Herkolim* variety [25], very similar contents and profiles are observed with respect to the *Primens* variety studied here, except in the case of spear by-product, since here, the TDF NSP is slightly lower due to its minor glucose content. This fact may be related to the variety, the area of cultivation and the time of harvest. Insoluble fiber fraction (insoluble NSP) constitutes most of the total fiber in these samples. As in the case of total fiber, the highest content was found in the spear by-product (34.66 g/100 g), then the root by-product (26.44 g/100 g) and the lowest contribution was found in the edible portion of the spear (9.99 g/100 g). The ratio of neutral sugars/uronic acids, as in TDF NSP, was higher in the root (95/5), then in the basal part of the asparagus (91/9) and finally in the edible part (88/12), where there was quantified a greater presence, in proportion, of uronic acids. The monomer profile in IDF NSP was similar to that of TDF. In root and spear by-products, xylose and glucose were significantly higher than in the edible portion.

In spear edible portion there was a greater balance between the monomers although there was a greater presence of glucose. The composition of the SDF NSP was calculated by difference between TDF and IDF. The basal part presented the highest fiber content and the lowest proportion of uronic acids (25%) and, therefore, the greatest presence of neutral sugars (75%), among which glucose stood out, from which the importance of glycans in this area can be deduced. In the apical zone, soluble fiber consisted basically of uronic acids, with the presence of neutral sugars being less important. The soluble fiber from the root was quantitatively the smallest of the three fractions (2.94 g/100 g) and neutral sugars and uronic acids were in equal proportion. All the differences indicated were statistically significant (*p* < 0.05).

Villanueva-Suarez et al. [37] studied the dietary fiber of the edible part of white asparagus using an analytical methodology similar to that used in this work and presented a total NSP content of 21.68 g/100 g, insoluble NSP of 15.66 g/100 g and soluble NSP of 6.03 g/100 g. These data are higher than those obtained in this work, but it should be taken into account that, in addition to being a different variety and type of crop, the length of the edible part analyzed was 17–22 cm compared to 15 cm in the present work. The dietary fiber composition of the spear is different along the length of the spear, indicating the importance of the length of the area analyzed [25]. The insoluble fiber/soluble fiber ratio is 2.6, the same for the two types of asparagus. In the case of the work also carried out with white asparagus by Redondo-Cuenca et al. [38] the length of the spear was similar to that of [37], and the results obtained (15.58, 11.92 and 3.67 NSP total, insoluble and soluble g/100 g dry matter, respectively) are more similar to those of the present publication. This indicates that, in addition to the length of the spear analyzed, other factors such as the variety, type of crop and year of harvest also play a role.

The results obtained by applying the AOAC method (Table 1) were superior to those calculated by the sum of the constituent monomers (Table 3). This is because the two methods applied to determine fiber have different rationales. The AOAC method is based on hydrolyzing the components that are not part of the fiber, but the final measurement is gravimetric, i.e., the fiber is measured as a whole. The Englyst method performs a fiber isolation similar to the AOAC method but then hydrolyzes the non-starch polysaccharides (cellulose, hemicelluloses and pectins), and each monomer is determined. There may be other components, such as polyphenols, resistant protein, etc., that are quantified in the AOAC method but not in the Englyst method.

The presence of lignin was calculated based on the difference between the value of TDF and total NSP in the three fractions analyzed. The highest content for Klason lignin was found, as expected, in the spear by-product (14.60 g/100 g dry matter), as it is an older tissue that is characterized by being lignified; on the contrary, the apical area, which is the youngest part of the spear, had the lowest content (9.15 g/100 g). The root had an intermediate content (10.86 g/100 g). This can be explained because lignin is deposited in the cell walls of plants as part of the process of cell maturation after cell elongation has ceased [39].

If these results are compared with those presented by Redondo-Cuenca et al. [25] in green asparagus of the *Herkolim* variety, it can be observed that they were very similar to those of this work except in the case of the spear by-product, since the aforementioned authors showed lower lignin results. As previously mentioned, this area of the shoot is the most lignified due to the evolution of the tissue itself and may be influenced by different factors such as variety and harvest time.

### 3.4. Fructans and Phenolic Compounds

Asparagus contains many bioactive phytochemicals [28]. Among them, fructooligosaccharides and polyphenolic compounds stand out.

Table 4 shows the fructan values of the analyzed samples. Inulin was found in all of them, but only in the root was it possible to quantify small amounts of DP4 and DP3. In this same portion of the root, a significant amount of inulin was quantified (14.92 g/100 g) unlike the basal part of the spear (1.52 g/100 g) and the apical area (1.48 g/100 g). In asparagus, inulin is the reserve carbohydrate and is degraded by fructan exohydrolase (FEH) to provide energy for the emerging asparagus spear [40]. This may be the reason why the presence of inulin was so high in the root and decreased from the basal part to the apical part of the spear. Inulin has a clear prebiotic effect and its presence in the root by-product is very interesting because it could be used as a raw material for the extraction of this fructan. As Stribling & Ibrahim [41] indicated, oligosaccharides and inulin occur naturally in numerous foods and are frequently incorporated into commonly consumed food products for a variety of purposes, such as to increase dietary fiber content.

The asparagus samples studied presented high contents of polyphenolic compounds, reaching significantly higher values in the aerial parts, mainly in the hard stem portion. Polyphenolic compounds were quantified considering the degree of polymerization and grouped into low degree of polymerization polyphenols (LMP) and high degree of polymerization polyphenols (MP), differentiating between proanthocyanidins (MPP) and hydrolysable polyphenols (MHP). The three samples presented high polyphenol contents. In all cases, the MP content was higher. This is an important characteristic because the bioavailability of these bioactive compounds in the body and the consequent nutritional properties are different and therefore can have different repercussions on the health of the consumer. A significant part of LMP could be absorbed in the small intestine [13]. However, compounds with a high degree of polymerization cannot be absorbed in the small intestine. They reach the colon and interact with the colonic microbiota. As a consequence, non-absorbable active metabolites can be produced that remain in the colonic lumen, where they can interact with the colonic ecosystem, or the microbiota can break down the structure of MP and give rise to absorbable metabolites that reach target organs through blood. In this context, the intestinal microbiota plays a fundamental role in the metabolism of MP, although we should differentiate between MPP and MHP [40]. In addition, let us not forget that the interaction between polyphenolic compounds and the microbiota occurs in both directions; that is, the microbiota can degrade polyphenols and originate active metabolites, but in turn, both the original polyphenols in the sample and the metabolites formed can modify the bacterial population both in number and in bacterial species [42]. Obviously, the physiological consequences depend on the degree of intestinal bioaccessibility of each compound [43]. It is important to determine the bioavailability and intestinal bioaccessibility of the samples to try to come to more precise conclusions about the effects derived from their consumption. On the other hand, polyphenolic compounds are also related to texture changes in asparagus during postharvest storage due to chemical changes that occur in the cell wall structure. The aerial parts of the asparagus are richer in polyphenolic compounds, such as ferulic acid and hydroxycinnamic esters. These amounts may increase during the storage period [28]. In addition, asparagus also contains polysaccharides such as pectins, xylans and xyloglucans that could form polysaccharide–phenolic complexes in the asparagus cell wall, which lignify the pericycle of the plant, mainly in the basal zone [44].

The content of flavonoids, phenolic acids and saponins in the edible part of the spear is very low, so we would need to consume large quantities of asparagus to ingest the recommended dose of these bioactive compounds. In our previous studies on the valorization of asparagus by-products, it has been determined that these are an excellent source of bioactive compounds that are worth recovering, as their content is significantly higher in the by-products than in the edible portion of asparagus. Therefore, obtaining bioactive extracts enriched in phenols and/or saponins from the stems (spear by-product) or roots of asparagus is ideal for the development of nutraceutical formulations that are of great interest to consumers.

On the other hand, it is well known that the phytochemical profile of plant products is largely determined by genetic factors [45], which can lead to significant differences in both the qualitative and quantitative composition among different varieties and genotypes. In the case of asparagus spears, significant differences have been found in the profile of flavonoids [46] and saponins [18]. However, until now, it had not been investigated whether these differences also extend to other plant organs. In one of our recent publications, we addressed the phytochemical analysis of by-products derived from *A. officinalis, L. Herkolim* variety, and investigated their potential as modulators of the growth of human gut-associated bacteria [25]. The present work focuses on *A. officinalis*, *L. Primens* variety, from the same origin as the samples in the previous study, IFAPA Rancho de la Merced Center in Jerez (Chipiona, Cadiz, Spain). The results of the phytochemical composition analysis are shown in Table 5.

The qualitative composition of the samples from *Herkolim* and *Primens* was very similar, with a high content of flavonoid-like phenols in the edible part and a large amount of saponins in the root. The stems that are discarded as the final portion of the spear during processing contained moderate amounts of phenols and saponins. Nevertheless, significant differences have been found in the quantitative composition of the three types of samples. The phenolic content, mainly caffeic acid, of *Primens* root (130.70 ± 6.96 mg/100 g dry weight) was very similar to that previously reported for *Herkolim* (191.50 ± 5.71 mg/100 g dry weight). However, the latter had considerably higher amounts of saponins (1306.80 ± 63.20 mg/100 g dry weight) than *Primens* (953.17 ± 51.29 mg/100 g dry weight). In the spear bottoms from the *Primens* samples, 10 times more phenols (370.82 ± 29.26 mg/100 g dry weight) were quantified than saponins (38.27 ± 3.94 mg/100 g dry weight), which represents a significant difference compared to *Herkolim*, where the quantities of both phytochemicals were of the same order (138.00 ± 4.80 mg/100 g dry weight of flavonoids and 103.30 ± 3.70 mg/100 g dry weight of saponins. The spear edible portion also stands out for its high content of flavonoids, well above that of the varieties used for asparagus cultivation [47], and even superior to that of most wild species [48].

From the results obtained in this study, it can be noted that the differentiation of *Primens* samples from other asparagus varieties is based on a significantly higher content of phenolic compounds compared to most cultivated asparagus varieties and closer to that of wild varieties, which are known for their higher phytochemical contents.

It can be concluded that products and co-products from the *A. officinalis Primens* variety are of great interest for obtaining bioactive ingredients, such as flavonoids and saponins, with enormous potential for application in the field of food health, where there is a growing demand for natural extracts to replace chemically synthesized active ingredients, the use of which is subject to increasingly restrictive legislation.

### 3.5. Functional Properties

As previously described, the three parts of the asparagus studied could be considered as “high fiber”, according to the nutrition and health claim (Regulation (EC) No 1924/2006 of the European Parliament and of the Council of 20 December 2006 on nutrition and health claims made on foods. http://data.europa.eu/eli/reg/2006/1924/oj accessed on 15 January 2024), although the quantity and quality of each of them was different (Table 1 and Table 3) and these differences between them condition their functional and technological properties.

Many of the beneficial effects of fiber are related to its ability to increase fecal bulk and the rate of passage through the gastrointestinal tract. Most of the indigestible dietary components are largely related to the ability of fiber to retain water. Hydration properties are of paramount importance in predicting the transit time from mouth to colon and explaining many of the physiological effects of dietary fiber.

Hydration properties such as swelling and water retention capacity have been proposed to be of value to predict the ability of non-digestible dietary components to alter stool weight. These properties depend on the chemical composition and physical structure of fiber components. The chemical composition, the anatomy and the physical characteristics of the fibers and the type of processing influence the hydration properties and, consequentially, the behavior of fiber during gut transit. Furthermore, the range of application of these by-products as food ingredients is dependent, to a large extent, on their interaction with water and oil [49].

The hydration properties of asparagus samples are listed in Table 6. The swelling capacity of the root was the lowest, the hard stem reached intermediate values and the asparagus spear presented the highest swelling capacity. The differences between the three samples were statistically significant. They are probably more a function of fiber structure than chemical composition, since chemical analysis does not appear to change within each fiber source, whereas the structures and the WHC change. In this investigation, a quantitative determination of major chemical components of each sample has been made and compared with the WHC of each sample.

Water molecules hydrogen-bond to the exposed hydroxyl groups of amylose and amylopectin, causing an increase in WHC [50]. Furthermore, the carbohydrate composition also influences the hydration properties of the samples [51], since these constituents contain hydrophilic parts, as well as polar or charged side chains [52].

As previously mentioned, the sample of the edible portion of the asparagus contained a higher proportion of soluble fiber and a lower IDF/SDF ratio, and as can be seen in Table 6, this sample presented higher values from WHC and swelling.

These properties of dietary fiber are interesting both from the physiological and technological point of view, since in addition to the physiological effects on intestinal transit mentioned above, it must also be taken into account that samples with a high-water retention capacity could be good ingredients in applications that necessitate the addition of water, such as in the bakery industry.

The results obtained for the samples tested in this work are in the range of most described fibers, e.g., 11–20 mL water/g for fiber-rich powders from asparagus by-products [53] and 11 mL water/g for lemon fiber [54]. Other agricultural by-products had lower values, such as cocoa husks [55] and rice bran [56], both with a WHC value of 5 mL water/g.

On the other hand, oil absorption capacity is also an important functional property in food. OHC corresponds to the amount of oil that a sample can absorb per unit of weight. The asparagus samples’ capacity to retain oil was similar to that observed for the aerial samples and significantly lower for the root (Table 6). These values were higher than those published by other authors for asparagus by-products [57] and other materials such as unripe banana flour [58], indicating the possibility of using these by-products in products where emulsifying properties are required.

Oil retention is the result of binding the hydrocarbon chains of oil to the nonpolar compounds of the materials [59]. OHC is also essential in the manufacture of products such as doughnuts, pancakes, baked goods, desserts, confectioneries, beverages, salad dressings, meats extenders and meat analogues [51,60]. An ingredient with high OHC can be used to improve the texture of a product. It can also be used to improve the juiciness of meat products. It is possible that greater porosity of the aerial structures allows a greater retention of oil. These aspects should be studied further.

The water- and oil-holding capacities of materials play an important role in the food preparation process because they influence other functional and sensory properties. OHC is important since fats act as a flavor retainer and increases the mouth feel of food [61].

In relation to the ability of the samples to retain glucose, the glucose retardation index has been estimated (Table 6). A slower diffusion of glucose in the small intestine may be an important part of the action of certain types of dietary fiber in reducing the rate of glucose absorption. Therefore, reducing the speed of absorption in the small intestine of glucose from foods with glycemic carbohydrates, using fibers that show this ability to retain glucose, is of special importance in diets for the control of postprandial glycemia. The nature of polysaccharide determines its physico-chemical behavior, and this may affect the rate of digestion of carbohydrates and absorption of sugars in the small intestine.

The dialysate glucose concentrations for each other were all significantly different from each other (*p* < 0.001). The edible spear sample presented the highest values of the glucose release delay index (12.46 ± 0.26), followed by the spear by-product (8.39 ± 0.13) and the root, whose value for this index was very low (1.21 ± 0.04). As previously described in relation to the ability to retain water in the fiber matrix, glucose retention is also dependent on the components of the fiber matrix. It seems to be related to the SDF content and the uronic acid content of the IDF [62], although it may also be related to the internal structure and surface characteristics of the fibers. As can be seen in Table 3 and as was previously discussed, the fiber of the edible portion contained 27% of soluble components, while in the hard stem and root by-products, the soluble fiber only corresponded to 19% and 10%, respectively. Likewise, the uronic acid content (Table 3) was higher in the edible aerial part of the asparagus, which could explain the GRI values found in these samples (Table 6).

Despite the fact that the GRI of the edible portion presented the highest values in this study, it must be taken into account that it is much lower than those found in other fiber compounds, such as citrus pectins (31.50 [55]), apple pectins (34.90 [63]) or guar gum (41.01 [63]), that are frequently used as ingredients in foods intended for glycemic control.

In summary, the aerial samples of asparagus showed a greater capacity to retain water, oil and glucose, so both the spear byproduct and the spear edible portion could be appropriate to use as functional ingredients in fiber-enriched foods that require emulsification and/or retain water.

All these properties of the samples studied seem to be related to the composition of the fiber fraction contained in them, as indicated by the statistical analysis. The Pearson correlation coefficient was used to compare each of the properties studied with TDF in the part of the asparagus studied. Thus, the swelling presented a strong direct correlation for the TDF of the spear by-product and edible spear (R2 = −0.9841) and a weak inverse correlation for the root by-product. When studying the correlations for the WHC, the TDF showed direct correlation for the edible part, being inverse for the rest of the fractions. The OHC could not be found to be correlated in TDF for the root and was confirmed as inverse and strong for the other fractions. As far as GRI is concerned, strong correlations were found for TDF, direct for spear by-product and edible part (R2 = 0.9724) and inverse for the root (R2 = −0.9596).

### 3.6. Impact of the Asparagus Fractions on Bacterial Growth

The results describing the effect of the different asparagus parts on the growth of the indicator organisms are shown in Table 7 and Table 8. In the case of the probiotic strains (Table 7), the three fractions were able to promote their growth although, overall, the growth-promoting effect of the edible spear was significantly higher and broader in comparison to both the edible spear and the root. However, the positive effect of the edible and non-edible spears was similar for the *L. reuteri*, *L. rhamnosus* and *B. longum* strains and for one of the two *B. breve* strains tested in this work.

In contrast, the growth of the pathobionts strains was not promoted by any asparagus fraction. No differences were found between the growth in root-supplemented cultures and the respective controls for any pathobiont strain (Table 8). However, the counts in all the cultures containing either the edible or the non-edible spear were significantly lower than those found in the respective controls (Table 8). The only exception was *Salmonella typhimurium* MP399, since there was no difference between the cultures supplemented with the non-edible spear and the controls.

**Figure 1:**
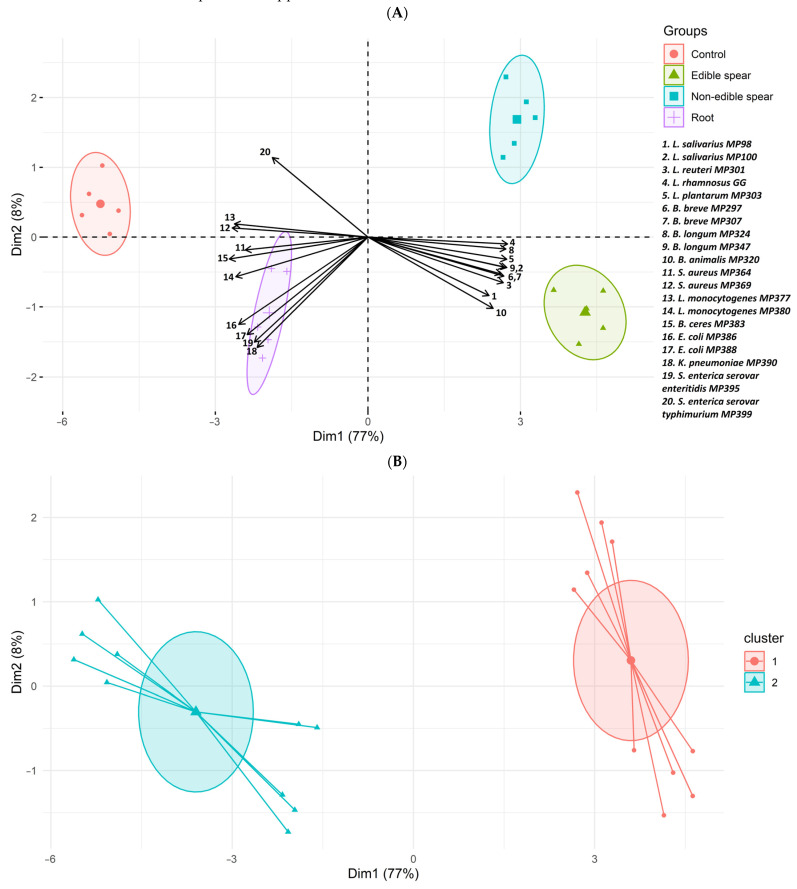
Principal component analysis (PCA) of culture samples from the edible, non-edible, root asparagus fractions and the control group. Each point in the plot represents an individual sample and different colors are used to distinguish between different asparagus fractions (**A**). Confidence ellipses encompassing 75% of the samples within each respective cluster are also included. The clustering analysis (**B**) reveals two significant clusters. The first cluster, prevalent in control and root sections, exhibits a positive correlation with pathogenic bacteria. In contrast, the second cluster, predominantly found in spear by-product and edible portions, shows a positive correlation with probiotic bacteria.

The principal component analysis (PCA) applied to the dataset revealed the existence of two principal components (PC) that accounted for 85% of the total variance (PC1: 77%, PC2: 8%). The first principal component (PC1) had significant contributions from the *L. salivarius* MP100, *L. rhamnosus* GG and *L. plantarum* MP303 strains, while the second principal component (PC2) largely influenced *E. coli* MP388 and *K. pneumoniae* MP390 strains (Figure 1A).

Further cluster analysis revealed two distinct clusters (Figure 1B), with one cluster including the samples from the control and the root-containing cultures, and the second cluster containing the spears (by-product and edible) fractions. Interestingly, these clusters were associated with different bacterial profiles. The first cluster showed a positive correlation with pathobionts, indicating a higher abundance of these bacteria in the control and root-related samples. In contrast, the second cluster displayed a positive correlation with probiotic bacteria, suggesting the higher impact of both spear fractions on the growth of the beneficial bacterial strains included in this work.

The fact that the three fractions somehow stimulated the growth of the *lactobacilli* and *bifidobacteria* strains may be explained on the basis of their richness in some compounds that can be used by these either in a non-selective (e.g., free sugars) or in a selective (e.g., fructans) manner. Such bacterial groups are able to produce a wide variety of enzymes, enabling the use of many carbohydrates by specialized metabolic pathways. The different proportions of some compounds in the three parts of the asparagus may explain why the effects of the non-edible and edible spears (with higher content in fructose, glucose and polyphenolic compounds) were more pronounced than that of the root fraction. The spatial distribution (from the root to the edible spear) of some compounds in the asparagus has been previously reported [32,41]. In addition, polyphenolic compounds have the potential to act as prebiotics, promoting the selective growth of the *lactobacilli* and *bifidobacteria* [64,65,66] while restraining that of pathogen strains [67,68,69,70].

The results of this work regarding the impact of the three asparagus fractions on some bacterial strains are similar to those obtained recently for another asparagus variety, *Herkolim,* using the same methodology, although some differences also exist between them [25]. Overall, the effects of the *Herkolim* fractions (increasing the growth of probiotic bacteria and controlling that of pathobiont bacteria) seem to be somehow stronger than those of the respective *Primens* fractions analyzed in this work. A previous study has revealed significant differences in the composition of the root fractions of these two varieties (e.g., a higher percentage of fructans in *Herkolim*) [71], a fact that may account for the slight differences observed when comparing the two varieties. Regardless, the results of this work show that the edible spear of the *Primens* variety has the potential to provide beneficial effects to the composition of the gut microbiota when ingested as a part of the diet, while the non-edible spear and the roots may also be employed as a source of natural prebiotic supplements.

## 4. Conclusions

The edible part and the stem and root by-products of green asparagus, var. *Primens*, presented a high content of nutrients; bioactive polyphenolic compounds, mainly MP; and substantial amounts of inulin and other non-digestible carbohydrates. All three asparagus fractions were able to promote the growth of probiotic strains, but not that of pathogenic strains. The effect was more marked in the aerial samples and seems to be related to the composition of the non-digestible fraction of the samples. In addition, the samples showed good water and oil retention capacity and discrete GRI values. In summary, according to the results, all fractions of the *Primens* variety have a composition with potential health benefits and functional properties of interest. The edible stem has the potential to provide beneficial effects to the gut microbiota composition when ingested as part of the diet, while the non-edible stem and roots could also be used as raw material for fructan extraction or as a source of natural prebiotic supplements with physiological effects on intestinal transit. In addition, asparagus by-products are of technological interest because they could be used as functional ingredients in fiber-enriched foods that require emulsification and/or retain water.

## Figures and Tables

**Table 1:** Proximal composition of green *Asparagus officinalis* L., var. *Primens* (root by-product, spear by-product and spear edible portion). Results are expressed as g/100 g dry matter ^1^.

	Root by-Product	Spear by-Product	Spear Edible Portion
Protein	17.80 ± 0.03 ^b^	14.96 ± 0.54 ^a^	48.82 ± 0.23 ^c^
Fat	1.00 ± 0.13 ^a^	2.08 ± 0.06 ^b^	3.39 ± 0.76 ^c^
Total Carbohydrates	32.11 ± 0.80 ^c^	28.68 ± 0.88 ^b^	17.27 ± 0.29 ^a^
Sucrose	2.09 ± 0.24 ^b^	0.24 ± 0.06 ^a^	0.28 ± 0.00 ^a^
Glucose	1.00 ± 0.01 ^a^	5.41 ± 0.23 ^b^	5.69 ± 0.00 ^b^
Fructose	1.68 ± 0.01 ^a^	9.42 ± 0.69 ^b^	9.04 ± 0.00 ^b^
Total dietary fiber	40.52 ± 0.61 ^b^	57.70 ± 0.57 ^c^	22.85 ± 1.00 ^a^
Ash	5.01 ± 0.20 ^a^	4.87 ± 0.05 ^a^	9.34 ± 0.01 ^b^

^1^ Data are the mean of ≥3 determinations ± SD. Values followed by different superscript letter significantly differ (*p* < 0.05).

**Table 2:** Contents of macro- and microelements of green *Asparagus officinalis* L., var. *Primens* (root by-product, spear by-product and spear edible portion). Results are expressed as g/100 g or mg/100 g dry matter ^1^.

Element	Root by-Product	Spear by-Product	Spear Edible Portion
K (g/100 g)	1.49 ± 0.16 ^a^	2.32 ± 0.17 ^b^	2.70 ± 0.03 ^c^
Na (g/100 g)	0.05 ± 0.01 ^b^	0.02 ± 0.0 ^a^	0.08 ± 0.02 ^b^
Ca (g/100 g)	0.35 ± 0.03 ^c^	0.15 ± 0.00 ^a^	0.23 ± 0.00 ^b^
Mg (g/100 g)	0.11 ± 0.01 ^b^	0.05 ± 0.01 ^a^	0.21 ± 0.01 ^c^
Fe (mg/100 g)	0.03 ± 0.00 ^b^	0.01 ± 0.00 ^a^	0.01 ± 0.00 ^a^
Zn (mg/100 g)	0.01 ± 0.00	nd	nd
Cu (mg/100 g)	nd	nd	nd
Mn (mg/100 g)	nd	nd	nd
Ash (g/100 g)	5.01 ± 0.20 a	4.87 ± 0.05 a	9.34 ± 0.01 b

^1^ Data are the mean of ≥3 determinations ± SD. Values followed by different superscript letter significantly differ (*p* < 0.05). nd: not detected.

**Table 3:** Dietary fiber: neutral sugars and uronic acids of green *Asparagus officinalis* L., var. *Primens* (root by-product, spear by-product and spear edible portion). Results are expressed as g/100 g dry matter ^1^.

	Root by-Product	Spear by-Product	Spear Edible Portion
**TDF neutral sugars**			
Arabinose	1.63 ± 0.01 ^c^	0.52 ± 0.03 ^a^	0.98 ± 0.01 ^b^
Xylose	6.45 ± 0.07 ^b^	14.07 ± 0.11 ^c^	1.16 ± 0.03 ^a^
Mannose	0.40 ± 0.02 ^a^	0.46 ± 0.01 ^b^	0.41 ± 0.02 ^a^
Galactose	0.70 ± 0.03 ^c^	0.65 ± 0.01 ^b^	0.52 ± 0.02 ^a^
Glucose	17.43 ± 0.22 ^b^	22.37 ± 0.27 ^c^	6.77 ± 0.22 ^a^
Total	26.61 ± 0.25 ^b^	38.07 ± 0.14 ^c^	9.84 ± 0.27 ^a^
**TDF uronic acids**	2.95 ± 0.01 ^a^	5.03 ± 0.01 ^c^	3.86 ± 0.01 ^b^
**TDF NSP**	29.66 ± 0.23 ^b^	43.10 ± 0.12 ^c^	13.70 ± 0.27 ^a^
**IDF neutral sugars**			
Arabinose	1.41 ± 0.01 ^c^	0.28 ± 0.03 ^a^	0.36 ± 0.00 ^b^
Xylose	6.37 ± 0.45 ^b^	12.28 ± 0.17 ^c^	1.01 ± 0.10 ^a^
Mannose	0.00 ± 0.00	0.23 ± 0.00 ^a^	0.41 ± 0.01 ^b^
Galactose	0.61 ± 0.03 ^b^	0.32 ± 0.01 ^a^	0.33 ± 0.01 ^a^
Glucose	16.60 ± 0.55 ^b^	18.53 ± 0.63 ^c^	6.67 ± 0.21 ^a^
Total	25.00 ± 0.09 ^b^	31.63 ± 0.76 ^c^	8.77 ± 0.11 ^a^
**IDF uronic acids**	1.44 ± 0.15 ^b^	3.03 ± 0.22 ^c^	1.22 ± 0.01 ^a^
**IDF NSP**	26.44 ± 0.05 ^b^	34.66 ± 0.91 ^c^	9.99 ± 0.11 ^a^
**SDF neutral sugars**			
Arabinose	0.22 ± 0.01 ^a^	0.29 ± 0.00 ^b^	0.63 ± 0.01 ^c^
Xylose	0.28 ± 0.17 ^a^	0.00 ± 0.00	0.15 ± 0.12 ^a^
Mannose	0.40 ± 0.02 ^b^	0.23 ± 0.01 ^a^	0.00 ± 0.00
Galactose	0.09 ± 0.03 ^a^	0.33 ± 0.01 ^c^	0.18 ± 0.02 ^b^
Glucose	0.45 ± 0.16 ^b^	4.52 ± 0.23 ^c^	0.11 ± 0.01 ^a^
Total	1.43 ± 0.34 ^a^	6.01 ± 0.47 ^b^	1.07 ± 0.24 ^a^
**SDF uronic acids**	1.51 ± 0.08 ^a^	2.00 ± 0.21 ^b^	2.64 ± 0.01 ^c^
**SDF NSP**	2.94 ± 0.18 ^a^	8.01 ± 0.45 ^c^	3.71 ± 0.23 ^b^

^1^ Data are the mean of ≥3 determinations ± SD. Values followed by different superscript letter significantly differ (*p* < 0.05).

**Table 4:** Other non-digestible components of green *Asparagus officinalis* L., var. *Primens* (root by-product, spear by-product and spear edible portion). Results are expressed as g/100 g dry matter ^1^.

	Root by-Product	Spear by-Product	Spear Edible Portion
Fructans			
Inulin	14.92 ± 0.15 ^b^	1.52 ± 0.16 ^a^	1.48 ± 0.17 ^a^
DP4	0.95 ± 0.01	0	0
DP3	0.84 ± 0.01	0	0
**Phenolic Compounds ^2,3^**			
LMP (extractable polyphenols)	0.99 ± 0.03 ^a^	1.79 ± 0.15 ^c^	1.15 ± 0.01 ^b^
MP (non-extractable polyphenols)			
MPP	0.56 ± 0.02 ^b^	0.43 ± 0.01 ^a^	0.64 ± 0.10 ^b^
MHP	1.44 ± 0.02 ^b^	1.63 ± 0.07 ^c^	0.61 ± 0.02 ^a^

^1^ Data are the mean of 3 determinations ± SD. Values followed by different superscript letter significantly differ (*p* < 0.05). ^2^ LP—low-molecular polyphenols; MP—macromolecular polyphenols; MPP—macromolecular proanthocyanidins polyphenols; MHP—macromolecular hydrolysable polyphenols. ^3^ The results are expressed as mg of gallic acid equivalents per g of dry matter.

**Table 5:** Flavonoids, phenolic acids and saponins of green *Asparagus officinalis* L., var. *Primens* (root by-product, spear by-product and spear edible portion). Results are expressed as mg/100 g dry matter.

		Root by-Product	Spear by-Product	Spear Edible Portion
**Flavonoids**			**370.82 ± 29.26**	**1534.13 ± 25.19**
Quercetin-triglycoside			41.49 ± 2.52	73.12 ± 1.85
Isorhamnetin-triglycoside			16.70 ± 1.12	2.91 ± 0.36
Quercetin-3-O-rhamnoglucoside (Rutin)			224.31 ± 19.41	1361.70 ± 16.21
Quercetin-3-O-glucoside			77.75 ± 5.29	53.65 ± 6.95
Kaemferol-3-O-rhamnoglucoside (Nicotiflorin)			2.72 ± 0.19	19.53 ± 0.43
Isorhamnetin-3-O-rhamnoglucoside (Narcisin)			7.85 ± 1.11	23.21 ± 0.25
**Phenolic acids**		**130.70 ± 6.96**	**0.96 ± 0.11**	**-**
Caffeic acid		130.70 ± 6.96	0.96 ± 0.11	
**Saponins**		**953.17 ± 51.29**	**38.27 ± 3.94**	**-**
[M-H]^−^	Previously described as			
1051.5	HTSAP1	423.27 ± 54.04		
919.5	HTSAP2	193.21 ± 32.63		
1047.7	Protodioscin	-	38.27 ± 3.94	
1048.6		53.62 ± 8.71		
1035.5	HTSAP6	55.52 ± 6.68		
1033.6		47.13 ± 6.73		
1043.6		26.41 ± 9.56		
755.6		119.74 ± 19.22		

**Table 6:** Functional properties of green *Asparagus Officinalis* L., var. *Primens* (root by-product, spear by-product and spear edible portion) ^1^.

	Root by-Product	Spear by-Product	Spear Edible Portion
Swelling (water mL/g dry matter)	1.62 ± 0.26 ^a^	7.07 ± 1.60 ^b^	12.27 ± 1.37 ^c^
Water-holding capacity (g/g dry matter)	3.61 ± 0.13 ^a^	9.72 ± 0.23 ^b^	12.71 ± 0.66 ^c^
Fat holding capacity (g/g dry matter)	2.82 ± 0.18 ^a^	5.86 ± 0.70 ^b^	5.53 ± 0.31 ^b^
Glucose retardation index	1.21 ± 0.04 ^a^	8.39 ± 0.13 ^b^	12.46 ± 0.26 ^c^

^1^ Data are the mean of 3 determinations ± SD. Values followed by different superscript letters significantly differ (*p* < 0.05).

**Table 7:** Effect of the different green *Asparagus officinalis* L., var. *Primens* fractions (root by-product, spear by-product and spear edible portion) on the growth of the probiotic strains included in this study. Bacterial counts are expressed as mean log_10_ CFU/mL ± SD. Each assay was performed in quintuplicate ^1^.

Strain	Control	Root by-Product	Spear by-Product	Spear Edible Portion	*p*-Value
*L. salivarius MP98*	9.40 ± 0.16 ^c^	9.86 ± 0.17 ^b^	9.92 ± 0.08 ^b^	10.22 ± 0.08 ^a^	<0.001
*L. salivarius MP100*	9.18 ± 0.08 ^d^	9.72 ± 0.08 ^c^	9.94 ± 0.05 ^b^	10.10 ± 0.07 ^a^	<0.001
*L. reuteri MP301*	9.22 ± 0.08 ^c^	9.62 ± 0.08 ^b^	9.78 ± 0.08 ^a^	9.86 ± 0.09 ^a^	<0.001
*L. rhamnosus GG*	9.20 ± 0.10 ^c^	9.52 ± 0.08 ^b^	9.82 ± 0.08 ^a^	9.94 ± 0.11 ^a^	<0.001
*L. plantarum MP303*	9.24 ± 0.11 ^d^	9.46 ± 0.11 ^c^	9.74 ± 0.05 ^b^	9.96 ± 0.09 ^a^	<0.001
*B. breve MP297*	8.82 ± 0.08 ^c^	9.10 ± 0.07 ^b^	9.22 ± 0.08 ^b^	9.36 ± 0.05 ^a^	<0.001
*B. breve MP307*	8.74 ± 0.13 ^c^	9.08 ± 0.11 ^b^	9.24 ± 0.05 ^ab^	9.4 ± 0.07 ^a^	<0.001
*B. longum MP324*	8.56 ± 0.11 ^c^	8.90 ± 0.12 ^b^	9.20 ± 0.07 ^a^	9.28 ± 0.11 ^a^	<0.001
*B. longum MP347*	8.58 ±0. 13 ^c^	9.04 ± 0.09 ^b^	9.22 ± 0.08 ^a^	9.38 ± 0.08 ^a^	<0.001
*B. animalis MP320*	9.00 ± 0.12 ^c^	9.14 ± 0.09 ^bc^	9.24 ± 0.09 ^ab^	9.38 ± 0.08 ^a^	<0.001

^1^ Different letters indicate significant differences among asparagus sections according to Tukey’s post hoc test (*p* < 0.05). An analysis of variance (ANOVA) was conducted to determine overall differences among sections, followed by Tukey’s test for multiple comparisons.

**Table 8:** Effect of the different green *Asparagus officinalis* L., var. *Primens* fractions (root by-product, spear by-product and spear edible portion) on the growth of the pathobiont strains included in this study. Bacterial counts are expressed as mean log10 CFU/mL ± SD. Each assay was performed in quintuplicate ^1^.

Strain	Control	Root by-Product	Spear by-Product	Spear Edible Portion	*p*-Value
*S. aureus* MP364	10.24 ± 0.11 ^a^	10.12 ± 0.16 ^a^	9.86 ± 0.11 ^b^	9.82 ± 0.15 ^b^	<0.001
*S. aureus* MP369	10.24 ± 0.11 ^a^	10.10 ± 0.16 ^a^	9.68 ± 0.101 ^b^	9.44 ± 0.11 ^c^	<0.001
*L. monocytogenes* MP377	9.20 ± 0.17 ^a^	9.10 ± 0.07 ^a^	8.82 ± 0.08 ^b^	8.62 ± 0.08 ^c^	<0.001
*L. monocytogenes* MP380	9.20 ± 0.10 ^a^	9.18 ± 0.18 ^a^	8.64 ± 0.17 ^b^	8.52 ± 0.13 ^b^	<0.001
*B. cereus* MP383	9.38 ± 0.08 ^a^	9.28 ± 0.19 ^a^	8.52 ± 0.13 ^b^	8.32 ± 0.13 ^b^	<0.001
*E. coli* MP386	9.36 ± 0.11 ^a^	9.24 ± 0.11 ^a^	8.46 ± 0.15 ^b^	8.74 ± 0.11 ^c^	<0.001
*E. coli* MP388	9.32 ± 0.16 ^a^	9.32 ± 0.08 ^a^	8.40 ± 0.07 ^b^	8.78 ± 0.08 ^c^	<0.001
*K. pneumoniae* MP390	9.08 ± 0.08 ^a^	9.06 ± 0.09 ^a^	8.40 ± 0.16 ^b^	8.74 ± 0.11 ^c^	<0.001
*S. enterica enteritidis* MP395	9.20 ± 0.12 ^a^	9.16 ± 0.19 ^a^	8.54 ± 0.09 ^b^	8.82 ± 0.08 ^c^	<0.001
*S. enterica typhimurium* MP399	9.24 ± 0.15 ^a^	9.20 ± 0.10 ^a^	9.08 ± 0.28 ^a^	8.66 ± 0.24 ^b^	0.001

^1^ Different letters indicate significant differences among asparagus sections according to Tukey’s post hoc test (*p* < 0.05). An analysis of variance (ANOVA) was conducted to determine overall differences among sections, followed by Tukey’s test for multiple comparisons.

## Data Availability

The original contributions presented in the study are included in the article, further inquiries can be directed to the corresponding author.
